# A rare cause of stroke: fail-implanted venous port catheter system – a case report

**DOI:** 10.1186/s12883-021-02191-y

**Published:** 2021-04-14

**Authors:** Mesut Yeniguen, Tobias Braun, Alexander Vlazak, Thomas Umscheid, Martin Juenemann, Tibo Gerriets, Marlene Tschernatsch

**Affiliations:** 1 Department of Neurology, University Hospital Giessen and Marburg, Klinikstrasse 33, 35392 Giessen, Germany; 2 Department of Neurology, Buergerhospital Friedberg, Ockstaedter Strasse 3-5, 61169 Friedberg, Germany; 3 HELIOS Klinik für Gefäßmedizin, Emser Straße 29-31, 65307 Bad Schwalbach, Germany

**Keywords:** Cerebral infarction, Port catheter system, Thrombemboly, Case report

## Abstract

**Background:**

We present the case of a 75-year-old female with acute embolic cerebral infarction caused by a fail-implanted venous port catheter system in the left subclavian artery.

**Case presentation:**

A 75-year-old woman presented to our emergency room after acute onset of a right-sided hemiparesis and dysarthria. Within 2 days after admission, she developed a left-sided hemiparesis, ataxia with concordant gait disturbance and incoordination of the left upper limb. DWI-MRI showed acute multiple infarcts in both cerebral and cerebellar hemispheres. Laboratory examination, 24-h Holter electrocardiography and transthoracic echocardiography provided no pathological findings. Further examination revealed an arterially fail-implanted port catheter, placed in the left subclavian artery with its tip overlying the ascending aorta, as the source of cerebral embolism.

**Conclusion:**

This is the first case report of thromboembolic, cerebral infarction due to a misplaced venous port catheter in the subclavian artery, emphasizing the imperative need for a thorough diagnostic workup, when embolism is suspected but cannot be proven at first glance.

## Background

Central venous port systems are widely used in modern medicine. The usual complications comprise of local or systemic catheter-related infections, local thromboses with swelling of extremities and malfunction due to mechanical abnormalities. Careful insertion and standardized procedures to ensure correct placement of the port catheter system are vital to avoid periprocedural complications.

We present the first case of a woman with cerebral infarction caused by a failed implanted venous port catheter system residing in the subclavian artery.

## Case presentation

A female in her 70s was admitted to our hospital with the suspected diagnosis of acute ischemic stroke after acute onset of a right-sided hemiparesis. Amongst others, the medical history revealed a short bowel syndrome after ascendojejunostomy with the necessity of diet therapy and parenteral nutritional supplementation via venous port catheter. The percutaneous implantation of the venous port catheter in the left subclavian vein was performed in a different hospital a week before hospital admission. Over recent years, several ports had already been removed due to catheter-associated complications like lumen occlusion and port-related infections. The port currently inserted had not been used for parenteral nutrition previously.

The patient underwent a standard acute stroke diagnostic workup consisting of non-contrast cranial computed tomography (CT), a 24-h Holter electrocardiography without indications for atrial fibrillation and transthoracic echocardiography revealing normal heart structures. The laboratory results from the patients blood were all within normal paramters, especially the coagulation test (INR, PTT). Extracranial and intracranial duplex sonography indicated marginal atherosclerosis without hemodynamically effective stenoses.

Secondary prophylaxis with antiplatelet (aspirin) and statin medication was initiated. Low-dose enoxaparin was also prescribed to minimise the risk of venous thrombosis. The patient benefited considerably from regularly performed physical therapy, and arm function improved during the first 48 h. Two days after admission, the patient showed an acute onset weakness of the left upper and lower limb, including mild cerebellar signs on the left side.

**Fig. 1:**
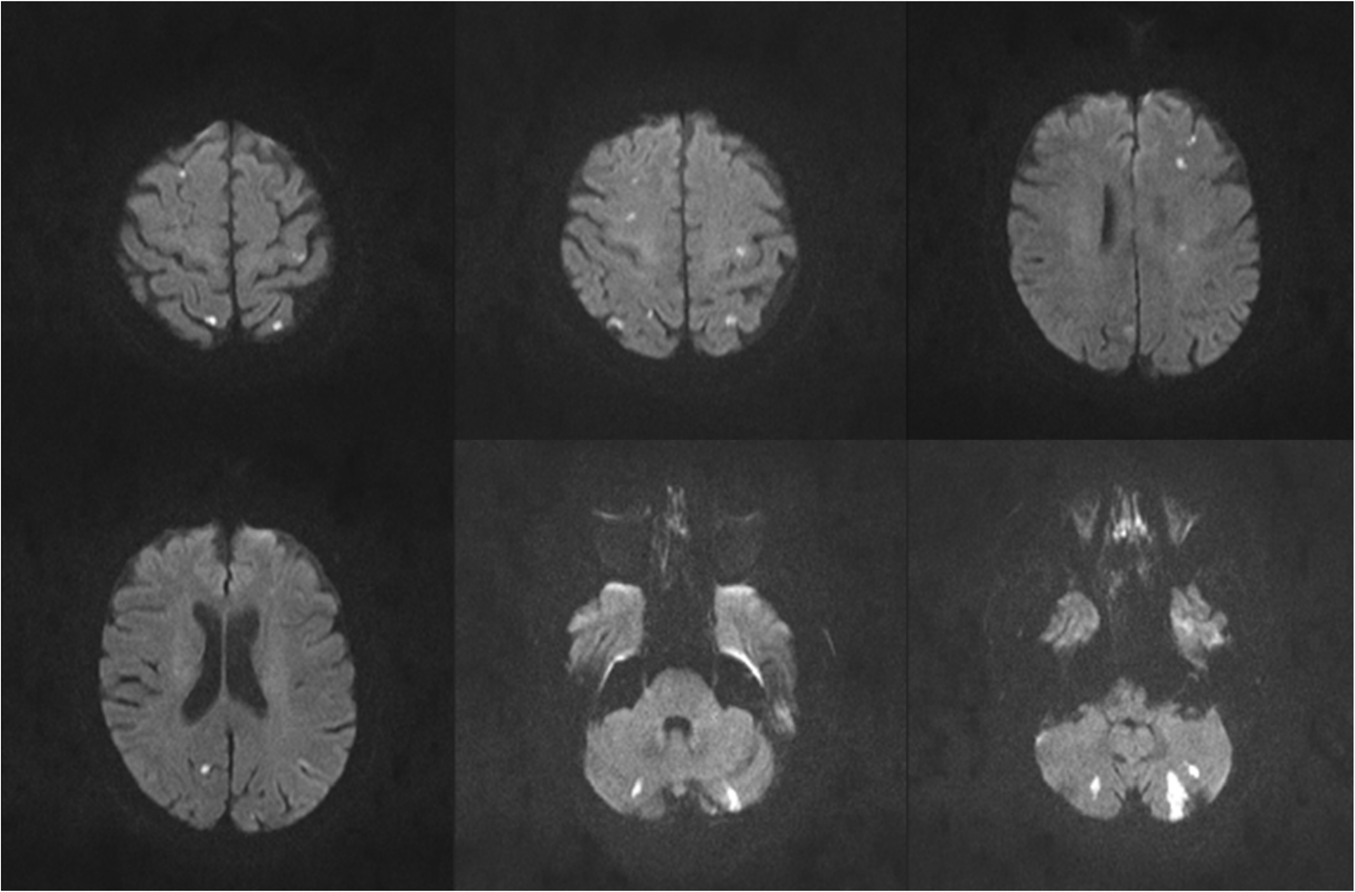
Axial DWI-MRI displaying hyperintense signals in both hemispheres in the cerebral cortex, subcortex and in the cerebellum

Brain magnetic resonance imaging (MRI) 2 days post stroke showed multiple small ischemic lesions bilaterally in the cerebellum as well as in subcortical and cortical areas of the cerebral hemispheres, affecting the supplying territory of arteries in the anterior and posterior circulation on both sides (Fig. 1). With regard to the cardioembolic pattern of the infarct lesions (Fig. 1) and - until this point - missing cardiac source of emboli, a bubble test by transcranial color-coded duplex sonography (TCCD) was performed, which couldn’t determine a right-to-left cardiac shunt.

Before injecting a small amount of saline solution into the port, the patency of the system was verified by cautious aspiration. As the blood returning to the syringe appeared moving pulsatile, an arterially misplaced port catheter was suspected.

**Fig. 2:**
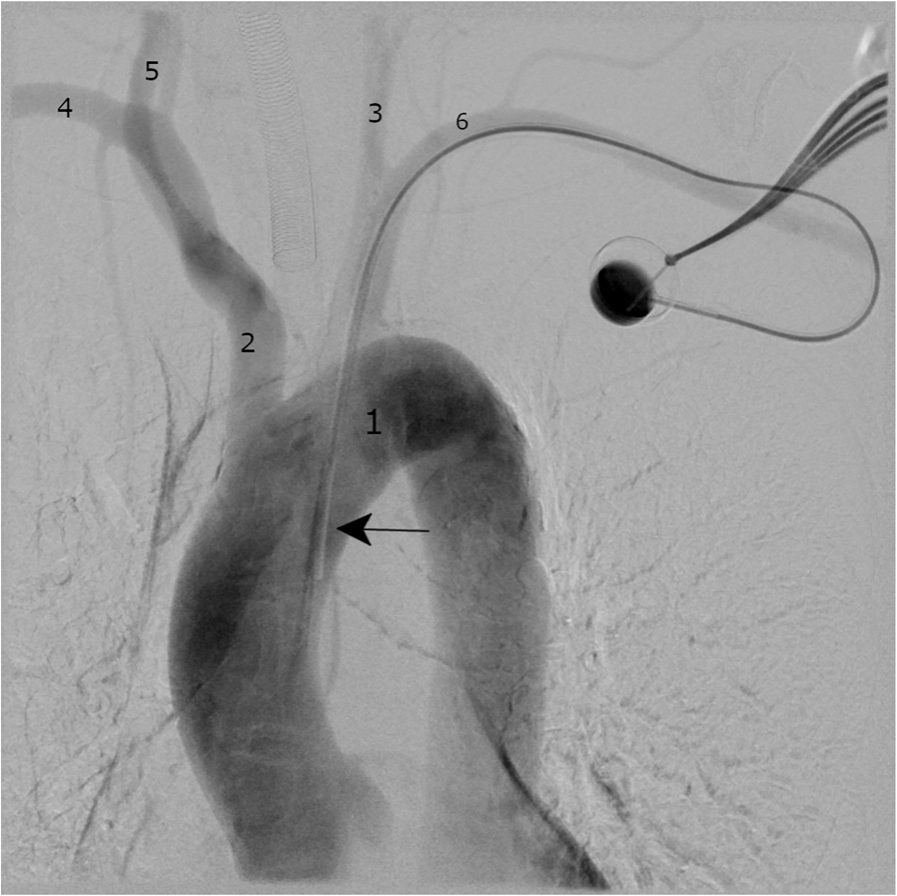
Intraoperative angiography of the aortic arch via port catheter system allows visualization of the misplaced port catheter and the supra-aortic arteries. Aortic arch (1), brachiocephalic trunk (2), left common carotid artery (3), right subclavian artery (4), right common carotid artery (5), left subclavian artery (6), tip of the catheter (arrow)

The patient was transferred immediately to a vascular surgery department. Conventional angiography showed an arterially placed catheter (Fig. 2) which was later removed during an open surgical exploration. Here, the catheter was found to be entering the left subclavian artery with an organized clot surrounding the catheter tip.

The patient’s neurologic status improved missing clinically overt signs of recurrent stroke, and she was able to be transferred to a rehabilitation center after implantation of a new central venous access port device. The patient recovered completely without any neurologic deficit 3 weeks after admission to our hospital.

## Discussion and conclusions

Central venous ports are considered a routine device for long-term administration of chemotherapeutic agents, transfusion blood sampling, repetitive antibiotics and intravenous hydration, or parenteral nutrition, mostly to cancer patients or—as in our case report—chronically ill patients [1, 2].

The use of a central venous port was initially described by Niederhuber et al., who reported the first implantation in 1982 via the subclavian vein [3].

A reliable implantation technique and regular maintenance of the venous port systems is indispensable in order to avoid catheter and port-related complications, such as catheter obstruction, venous thrombosis, infection and mechanical complications. In the literature referring to larger patient cohorts, the indicated complication rate ranges from 10.4 to 15.1% [4, 5]. In recent studies, an increased risk for ischemic stroke and cerebral gas embolism after implantation of a central nervous catheter were reported, which is associated with worse functional outcome [6, 7].

A broad variety of methods exist to implant a port catheter: Surgeons cut down or percutaneously puncture the forearm and upper-arm peripheral veins, the subclavian, or jugular vein, whereas radiologists perform minimally invasive venography, ultrasound, or fluoroscopy guidance of the subclavian or jugular vein [2, 8].

A careful insertion of the catheter system and skilled management should decrease the incidence of complications. The early diagnosis of arterial misplaced catheter can be obtained by the color of blood, the pressure and pulsatile backflow observed through the catheter, blood gas analysis and the postinsertion chest x-ray [2, 4, 8–10]. Obviously, the diagnosis of arterial misplacement of the catheter in the present case has been missed and not recognized during the insertion of the catheter, and the further routinely performed chest film following implantation.

Clinical findings and laboratory samples help to identify infectious complications, including local and catheter-related blood stream infections. Venous thrombotic complications usually manifest as arm or facial swelling, cyanosis, venous distension, and superficial venous collateralization. Mechanical complications include catheter malposition, occlusion or fragmentation, port damage and fibrin sheath formation. These complications lead to system malfunction, usually discovered earlier and, in consequence, system explantation and reimplantation may occasionally be required. Further complications, such as infusion pump dysfunction or human error, can also occur and are difficult to distinguish from mechanical complications [2, 9–11].

Such cases will have a rarity value. In current literature, no case has been described wherein an arterially displaced port catheter was left in situ a period of time, causing cerebrovascular events such as embolic cerebral infarction. Cerebrovascular symptoms caused by infusion of parenteral medication and nutrition via venous catheter were described only in a few cases [12].

Neurologic symptoms presented in temporal association with the insertion of a newly placed venous central port catheter might help to ensure the correct diagnosis before irreversible damage occurs. Intermittent embolization of small clots formed on the tip of the catheter following shear-induced platelet aggregation might be the possible pathophysiologic mechanism responsible for focal neurologic symptoms in the presented case. Nowadays, the arterially placed catheter would have probably been detected earlier, as CT-angiography is now routinely used in stroke patients, according to national stroke guidelines. This is due to the fact that CT-angiography helps to identify patients with large-vessel occlusion that might benefit from thrombectomy.

This case report highlights the symptoms of embolic cerebral infarction related to an arterially misplaced port catheter and emphasizes the imperative need for a thorough diagnostic workup when embolism is suspected but cannot be proven by standard diagnostic stroke workup.

### Take-home points

To avoid catastrophic complications of misplaced venous port symptoms, the following steps should be taken:

- Ultrasound guidance to localize the anatomic conditions of preferred vein
- Post insertion chest x-ray with critical evaluation
- Blood gas analysis and testing the catheter with about 100 ml of crystalloid solution running via gravity through newly placed catheter
- Reevaluation of catheter conditions in case of neurologic symptoms occurred with initiation of newly inserted catheter and after initiation of infusion.

## Data Availability

Data sharing is not applicable to this article as no datasets were generated or analysed during the current study.

## Abbreviations

CT: Computed tomography
DWI-MRI: Diffusion-weighted magnetic resonance imaging
MRI: Magnetic resonance imaging
TCCD: Transcranial color-coded duplex sonography
